# Glycoprotein 96 in Peritoneal Dialysis Effluent-Derived Extracellular Vesicles: A Tool for Evaluating Peritoneal Transport Properties and Inflammatory Status

**DOI:** 10.3389/fimmu.2022.824278

**Published:** 2022-02-10

**Authors:** Junyan Fang, Yan Tong, Ouyang Ji, Shan Wei, Zhihao Chen, Ahui Song, Pu Li, Yi Zhang, Huiping Zhang, Hongqiang Ruan, Feng Ding, Yingli Liu

**Affiliations:** ^1^ Division of Nephrology and Unit of Critical Nephrology, Shanghai Ninth People’s Hospital, School of Medicine, Shanghai Jiao Tong University, Shanghai, China; ^2^ Research and Development Center, Shanghai Applied Protein Technology Co., Ltd., Shanghai, China

**Keywords:** peritoneal dialysis, extracellular vesicles, glycoprotein 96, peritoneal solute transport rate, peritoneal inflammation

## Abstract

**Background:**

Extracellular vesicles (EVs) from peritoneal dialysis effluent (PDE), containing molecules such as proteins and microRNAs (miRNAs), may be potential biological markers to monitor peritoneal function or injury. Peritoneal inflammation is an important determinant of peritoneal solute transport rate (PSTR). Thus, the aim of this study is to determine whether the specific proteins capable of evaluating the PSTR could be found in PDE-EVs, and explore the underlying mechanism for the association between PSTR and peritoneal inflammation.

**Methods:**

Sixty patients undergoing peritoneal dialysis (PD) were divided into two groups: high/high average transport (H/A) group (PET >0.65) and low/low average transport (L/A) group (PET <0.65). EVs derived from PDE (PDE-EVs) were isolated by ultracentrifugation. Proteomic analysis was performed to explore the differentially expressed proteins and identify the potential biomarkers in PDE-EVs from the two groups, and we focused on glycoprotein 96 (GP96) as it could be involved in the inflammatory process. The expression of GP96 in PDE-EVs and inflammatory cytokines was quantified by real-time PCR and enzyme-linked immunosorbent assay. The infiltration of macrophages and neutrophils into the peritoneum was detected using immunohistochemistry in a PD rat model.

**Results:**

The expression of PDE-EVs-GP96 was significantly higher in the H/A group, and was positively correlated with the PSTR and the level of the inflammatory factor interleukin (IL)-6. GP96-enriched EVs enhanced the secretion of proinflammatory cytokines IL-1β, IL-6, tumor necrosis factor (TNF)-α, and IL-8 in macrophages, which was reversed by a pharmacological GP96-specific inhibitor (PU-WS13). The GP96 inhibitor also reduced local peritoneal inflammation by decreasing the infiltration of inflammatory cells and levels of proinflammatory cytokines (IL-6 and TNF-α) and chemokines (CCL2, CXCL1, and CXCL2) in a PD rat model.

**Conclusions:**

PDE-EVs-GP96 is a new promising tool to evaluate the status of peritoneal inflammation and PSTR, and the mechanism may be related to affecting the inflammatory properties of macrophages.

## Introduction

Patients with end-stage kidney disease undergoing peritoneal dialysis (PD) have vastly different capabilities for removing or transporting solutes and water *via* peritoneal membranes (1, 2). High peritoneal solute transport rate (PSTR) is associated with a greater decline in ultrafiltration (UF) volume and higher risks of technical failure, all-cause mortality, and hospitalization rates (3–5). The traditional PSTR measurement requires multiple collections of blood and drained peritoneal dialysate to calculate the 4-hour dialysate to plasma creatinine ratio (4 h D/P_Cr_). Intraperitoneal inflammation may be essential for peritoneal dysfunction in PD patients, and the PSTR reflects local inflammation within the peritoneal cavity (6). Several studies show strong evidence for potential associations between the PSTR and intraperitoneal interleukin (IL)-6 levels and peritoneal macrophage infiltration, which affects peritoneal dialysis outcomes (6–12). The multicenter GLOBAL study demonstrated that the dialysate IL-6 concentration is positively associated with the PSTR, but does not affect survival (7). Furthermore, IL-6 is not the only factor that affects the PSTR; genetic factors, effective peritoneal surface area, neoangiogenesis, endothelial cell functional injury, and peritoneal protein clearance rate also affect the PSTR (6, 12).

Extracellular vesicles (EVs), including exosomes, ectosomes, and apoptotic bodies, are nano- or micron-sized vesicles, that play an important role in intercellular communication, as they transfer biologically active molecules, including proteins, lipids, and nucleic acids, to target cells (13, 14). Recent studies have found EVs in the peritoneal dialysis effluent (PDE) that formed and accumulated in the peritoneal cavity during PD, possibly as a stress response (15, 17). Aquaporin-containing exosomes in PDE represent a potential non-invasive biomarker of dialysis efficiency (18). Carreras-Planella et al. determined that the EVs derived from PDE (PDE-EVs) proteome reveal alterations much earlier than peritoneal equilibration test (PET) monitoring (19), which suggests that proteins in the EVs derived from PDE are related to the PSTR. However, no study has investigated specific proteins capable of evaluating PSTR in PDE-EVs.

Substantial research has been undertaken to clarify the role of EVs in inflammation and inflammatory diseases. Proteomics is used to study EVs in inflammatory diseases, mainly to monitor changes in the expression or modification of proteins in normal versus pathologic states, which enables the identification of novel biomarkers and clarification of the underlying mechanisms in inflammatory diseases (20, 21). Although proteomic analysis of PDE has been performed in PD patients (22, 23), the biological characteristics and physiological roles of PDE-derived EVs remain unclear in patients with different PSTRs.

In this study, we used data-independent acquisition (DIA) technology for quantitative proteomics analysis, to explore the differentially expressed components in PDE-EVs from PD patients with different PSTRs, and to identify key proteins that were enriched in pathways of the immune inflammatory response. We hypothesized that GP96 may be a key protein associated with PSTR or peritoneal inflammation and verified this in clinical samples. Furthermore, we selected GP96-enriched EVs to stimulate macrophages *in vitro*, and then used a GP96 inhibitor to observe its role in inflammation. We also observed the effect of the GP96 inhibitor on peritoneal inflammatory cell infiltration and inflammatory cytokine secretion in a PD rat model. Collectively, our findings highlight PDE-EV-GP96 as a promising molecular marker to evaluate peritoneal transport characteristics and even a promising therapeutic target in peritoneal inflammation in PD patients.

## Materials and Methods

### Patients

We conducted a single-center study of 60 PD patients followed up at the Peritoneal Dialysis Center Department of Nephrology of Shanghai Ninth People’s Hospital. Inclusion criteria were as follows: (1) age 25–75 years, (2) had undergone regular PD therapy (defined as 3– 4 exchanges/day), (3) dialysis for >24 months, (4) serum albumin level ≥35 g/L, and (5) had been clinically stable for at least 3 months before the assessment. Patients with acute coronary syndromes, atrial fibrillation, severe valvular heart disease, cerebrovascular disorders, chronic lung disease, chronic liver diseases, infectious or non-infectious inflammatory diseases, malignancies, mental diseases, abuse of drugs or alcohol, pregnancy, and renal transplantation were excluded from the study.

Peritoneal transport characteristics were assessed using the criteria defined by Twardowski et al. (1). Patients underwent PET at 6-month intervals, and were classified into two transport categories based on their 3.86% 4 h D/P_Cr_, which were denoted as “high/high average (H/A, >0.65)” or “low/low average (L/A, <0.65).” The PSTR was considered to be stable if no changes in the PET results were detected three times in succession. We enrolled 60 patients with stable PSTRs and nocturnal dwell prescriptions in our study. Overnight peritoneal dialysate samples were collected after exchanging 2 L of PD solution (neutral pH, 2.27% glucose and lactate-buffered, Dianeal, Baxter Healthcare Corporation, USA) for 10 h. The dialysate was then drained and the remaining amount was measured.

Clinical data including age, sex, history of disease, use of medication, body mass index, dialysis duration, diabetes, hypertension, and 24-h UF volume were recorded. The causes of end-stage renal disease are mostly diagnosed based on clinical features.

### Isolation of PDE-EVs

The samples (500 mL of PDE per sample) were concentrated using a stirring UF cell using 10 kDa NMWL Ultracel regenerated cellulose membranes (EMD Millipore Corporation, USA) to obtain 40 mL of concentrated PDE per sample. The concentrate was centrifuged for 15 min (2500 ×*g*) at 4°C to remove cellular debris. After centrifugation for 30 min (18000 ×*g*) at 4°C, the supernatants were filtered through a 0.22-μm filter unit to remove larger particles, and then centrifuged for 2 h (120000 ×*g*) at 4°C using an ultracentrifuge (Optima XE-90, Beckman, USA) to obtain EVs. The remaining precipitate was washed with phosphate-buffered saline (PBS) and centrifuged at 120000 ×*g* for 2 h. The final pellets were then redissolved in 200 μL of PBS and stored at −80°C for further experiments.

### Transmission Electron Microscopy (TEM)

Transmission electron microscopy (FEI Tecnai F20, USA) was performed to identify the morphology of the obtained PDE-EVs. The PDE-EVs suspension was fixed in buffered 4% paraformaldehyde for 60 min at 4°C. Then, 10 μL of each sample was added to a formvar-coated copper grid and incubated for 10 min at room temperature. After washing with PBS, the grid was stained with 3% aqueous phosphotungstic acid for 2 min and finally evaluated using TEM at an acceleration voltage of 100 kV.

### Nanoparticle Tracking Analysis (NTA)

The size and concentration of PDE-EVs were measured by NTA (Zeta View, Germany), which is a combination of light scattering and Brownian motion technology. Each EV sample was diluted in pre-filtered PBS to an optimal concentration and then injected into the measurement room. The device performs measurements at 11 different locations in the measurement room and gives an average value. Each vesicle sample was measured three times to obtain the average value. Data were analyzed using ZetaView version 8.04.02.

### Mass Spectrometry-Based Proteomics Analysis

Single-pot, solid-phase-enhanced sample-preparation (SP3) technology was used to digest 10 H/A-EV and 10 L/A-EV samples. The detailed operational procedures have been described in our previous study (24). Briefly, after denaturation of the 50-μg EV proteins with lysis buffer, the proteins were reduced for 40 min with 10 mM dithiothreitol (DTT) at 37°C, alkylated with 25 mM iodoacetamide in the dark, and quenched with 50 mM DTT. The carboxylate-functionalized beads (1:10; μg of protein/μg of SP3 beads) and ethanol were added to achieve a specific final concentration (v/v 1:1), and then the tubes were incubated for 10 min at 900 rpm in a ThermoMixer. The supernatant was discarded after placement in a magnetic rack for 2 min, and the beads were rinsed three times with 180 μL of 80% ethanol. After washing, the beads were resuspended in 100 μL of 25 mM ammonium bicarbonate, trypsin (1:50) and Lys-C (1:100) were added, and the proteins were digested overnight at 37°C at 900 rpm. The supernatant was obtained using a magnetic rack, and then centrifuged at 4°C for 10 min. The last supernatant was desalted and dried by vacuum centrifugation. Before liquid chromatography-tandem mass spectrometry (LC-MS/MS), all samples were spiked with iRT peptides according to the manufacturer’s instructions (n=10 for each group).

The enriched peptides of all samples were analyzed by an in-house packed capillary column (75 μm i.d. × 25 cm, 1.9 μm/120-Å C18 resin) at 250 nL/min, and the gradient was as follows: 2 min, 5-8% B (0.1% (v/v) FA in 84% ACN); 80 min, 8-23% B; 26 min, 23-45% B; 2 min, 45-100% B; 10 min, 100% B. DIA (Data-dependent acquisition) technology was applied for the acquisition using a mass spectrometer (Q Exactive HFX) coupled with nanoLC1200 system (25, 26). The DIA method included 30 variable isolation windows and one full-scan window. The resolution of the full-scan and DIA scan resolution data was 120,000 and 30,000, respectively, the m/z range of full scan data was set at 350 to 1650 Da, and the normalized collision energy and automatic gain target of DIA scan were set at 25% and 3.0×10^6^, respectively.

Spectronaut 12.0 Pulsar (Biognosys) was used to analyze all raw files to generate an assay library for DIA analysis using default settings. The detailed information has been described in our previous study (24). Potential proteins were considered as differentially expressed between the groups when the fold change was ≥1.5 and *p*-value ≤0.05. For bioinformatic analyses, Blast2Go software (https://www.blast2go.com/) was applied to associate Gene Ontology (GO) terms with the differentially expressed proteins, and then functional enrichment analysis was performed using the online platform Database for Annotation, Visualization, and Annotated Discovery. The GO enrichment analyses consisted of three categories: biological process (BP), molecular function (MF), and cellular component (CC). All functional annotations were obtained from the UniProt database.

### Cell Culture, Treatment, and Uptake of EVs

Human monocytic THP-1 cells (THP-1) were obtained from ATCC (Manassas, USA) and cultured in RPMI 1640 medium (Gibco, Germany) containing 10% fetal bovine serum (Gibco) and 1% penicillin/streptomycin (Gibco). THP-1 monocytes were differentiated into macrophages by supplementing with 150 nM phorbol 12-myristate 13-acetate (Sigma-Aldrich, USA) over 24 h and starved in fresh RPMI 1640 medium overnight.

The effect of PDE-EVs on macrophage function was assessed to demonstrate the biological activity of GP96 in PDE-derived EVs. PDE-EVs were labeled with a PKH67 Green Fluorescent Cell Linker Mini Kit (Sigma-Aldrich, USA) according to the manufacturer’s protocol. Macrophages were incubated with PKH67 labeled EVs for 24 h and stained with 4′,6-diamidino-2-phenylindole (DAPI) to visualize the nuclei. Then, the labeled EVs were captured by living macrophages. The localization of EVs (green) and nuclei (blue) in the stained macrophages was observed using a fluorescence microscope to verify the uptake and internalization of the PDE-derived EVs.

The concentration of PDE-EV GP96 in the H/A and L/A groups was also quantified by an enzyme-linked immunosorbent assay (ELISA). Macrophages were incubated with H/A-PDE EVs (H/A-EVs, 2 mg/mL), L/A-PDE EVs (L/A-EVs, 2 mg/mL) (n=4), or H/A-PDE EVs combined with 0.5 μM PU-WS13 for 24 h, and changes in cytokine levels were measured by polymerase chain reactions (PCR) and western blotting. PBS was added to the control group.

### Cell Viability Assay

The macrophages were seeded into 96-well plates (1 × 10^4^ cells/well) and separately incubated with different PU-WS13 concentrations (0, 0.05, 0.5, and 5 µM) for 24 h. Then, 10 ul of Cell Counting Kit-8 (CCK-8) reagents (Biosharp, Shanghai, China) were added to each well for 2 h at 37°C. The absorbance was measured using a microplate reader (BioTek Epoch, USA) at 450 nm (*n* = 6 for each group).

### Animal Model and Experimental Groups

All rats received appropriate care in accordance with the animal protocols approved by the Animal Care and Use Committee of Shanghai Ninth People’s Hospital, Shanghai Jiao Tong University School of Medicine. All experiments were performed in accordance with the recommended guidelines. Thirty male Sprague-Dawley (SD) rats, weighing between 120 and 160 g, were obtained from the Chinese Academy of Sciences (Shanghai, China). All rats were housed with a 12 h light/dark cycle and had free access to food or water throughout the experiment. Animals were randomly divided into three groups: CKD rats (n=10), PD rats (n=10), and 17-AGG rats (n=10). All rats underwent a subtotal nephrectomy. Four weeks after subtotal nephrectomy, the rats underwent surgery to implant a peritoneal catheter connected to a subcutaneous mini access port for the acute model of exposure to 4.25% Dianeal (Baxter Healthcare Corporation, USA) or saline (5 mL/100 g body weight, twice a day for 10 days). The CKD group was instilled with saline. The PD group was instilled with peritoneal dialysis fluid (PDF). For 17-AGG experiments, PDF-instilled rats were treated with 17-AGG (50 mg/kg, Selleck Chemicals, Germany) every 3 days *via* the peritoneal catheter, starting 2 h before daily PDF instillation. After 10 days of follow-up, the animals were anesthetized using 9% chloral hydrate (0.3 mL/100 g, IM). Tissues from the peritoneum were collected and placed in 10% paraformaldehyde for immunohistochemical analysis or plunged into liquid nitrogen for PCR and western blotting.

### Immunohistochemical Staining

Peritoneum samples were collected and fixed in buffered 4% paraformaldehyde at room temperature, and then incubated for 1 h at room temperature in PBS containing 10% goat serum, 0.3 M glycine, 1% bovine serum albumin, and 0.1% Triton, which could permeabilize the cells and block non-specific protein-protein interactions. Then, peritoneum sections were treated with 3% H_2_O_2_ for 5 min at room temperature. The sections were incubated with primary antibodies specific for CD68 (1:1000; CST, USA) and MPO (1:2000; Abcam, United Kingdom) overnight at 4°C, followed by incubation with a horseradish peroxidase-conjugated anti-mouse secondary antibody for 60 min. Antibody binding was visualized using diaminobenzidine.

The number of macrophages or neutrophils was calculated by measuring the positive area of representative images (excluding anterior abdominal wall muscles) and analyzed using ImageJ (National Institutes of Health, USA).

### Western Blotting

Protein was extracted from isolated EVs with RIPA (Beyotime, China) and All-In-One buffers (Solarbio, China). A BCA Kit was used for protein quantification (Solarbio). Western blotting was performed as described in our previous study (24). Primary antibodies against CD9, CD81 (1:1000; SBI, USA), CD63, TSG101, CD31 (1:1000, Abcam, United Kingdom), mesothelin, and GP96 (1:1000; CST, United States) were used in this study. The protein density of each band was analyzed using ImageJ (National Institutes of Health, USA).

### Quantitative Real-Time PCR

Total RNA was extracted from the macrophages or peritoneum samples using TRIzol (Sigma), and then reverse transcribed into cDNA using Prime Script RT Master Mix (Takara, Japan). The cDNAs were subsequently used for gene expression by quantitative real-time PCR with the TB Green Premix Ex Taq kit (Takara, Japan). The PCR program was as follows: denaturation at 95°C for 30 s, followed by 40 cycles of 95°C for 5 s, and annealing at 60°C for 34 s. The primers used are listed in **Table 1**.

**Table 1:** Real-time PCR assays/sequences.

Gene	Assay or sequence
Homo sapiens	
IL-6	forward primer: ACTCACCTCTTCAGAACGAATTG
	reverse primer: CCATCTTTGGAAGGTTCAGGTTG
TNF-α	forward primer: CCTCTCTCTAATCAGCCCTCTG
	reverse primer: GAGGACCTGGGAGTAGATGAG
IL-1β	forward primer: ATGATGGCTTATTACAGTGGCAA
	reverse primer: GTCGGAGATTCGTAGCTGGA
IL-8	forward primer: GAAGTTTTTGAAGAGGGCTGAGA
	reverse primer: TTTGCTTGAAGTTTCACTGGCA
Rattus norvegicus	
IL-6	forward primer: CCTTCTCCACAAGCGCC
	reverse primer: TGCCTCTTTGCTGCTTTCAC
TNF-α	forward primer: CTCGAACCCCGAGTGACAAG
	reverse primer: TCAGCTTGAGGGTTTGCTACA
CXCL1	forward primer: CCCAAACCGAAGTCATAGCCA
	reverse primer: CGCCATCGGTGCAATCTATC
CXCL2	forward primer: ACCATCAGGGTACAGGGGTT
	reverse primer: CAACCCTTGGTAGGGTCGTC
CCL2	forward primer: TAGCATCCACGTGCTGTCTC
	reverse primer: GAGCTTGGTGACAAATACTACAGC

### Enzyme-Linked Immunosorbent Assay

PDE-EVs were extracted from 500 mL of PDE as described above. The concentration of GP96 in PDE-EVs was measured using commercial ELISA kits (Novus, USA). The minimum detected threshold concentration of GP96 protein was 0.31 ng/mL (n=20 for each group). PDE or cell culture suspension was centrifuged for 15 min at 2500 ×*g*. Then, the supernatant was collected and used for ELISA analysis of inflammatory cytokines. The cytokines, including TNF-α, IL-6, IL-1β, and IL-8, were examined using an ELISA kit (BioLegend, USA) according to the manufacturer’s recommendations. The minimum threshold concentrations of the TNF-α, IL-6, IL-1β, and IL-8 proteins detected by each ELISA kit were 15.6, 7.8, 2.0, and 15.6 pg/mL, respectively. Briefly, 100 µL of each sample or standard was added to the previously capture antibody-coated plate and incubated for 90 min at 37°C. After removing the liquid,100 µL of Biotinylated Detection Ab was added and incubated for 60 min at 37°C. After three washes, 100 µL of HRP conjugate was added to each well and incubated for 30 min at 37°C. After another three washes, 90 µL of substrate reagent was added and incubated for 15 min at 37°C. Then, 50 µL of stop solution was added and the absorbance was measured using a microplate reader (BioTek Epoch, USA).

### Statistical Analysis

Results are presented as the mean ± standard deviation (SD). Statistical differences were determined using an unpaired Student’s *t*-test for two groups, and one-way analysis of variance (ANOVA) for multiple groups. Spearman correlation was used for correlation analysis. All data analyses and graph generation were performed using IBM SPSS Statistics 23 (IBM Corp., Armonk, NY, USA) and GraphPad Prism 8.0.1 (GraphPad Software, San Diego, CA, USA). A *p*-value <0.05 was regarded as statistically significant. The enriched GO terms and Kyoto Encyclopedia of Genes and Genomes (KEGG) pathways were visualized using R-Studio software (RStudio, Boston, MA, USA).

## Results

### Characterization of PDE-EVs

**Figure 1:**
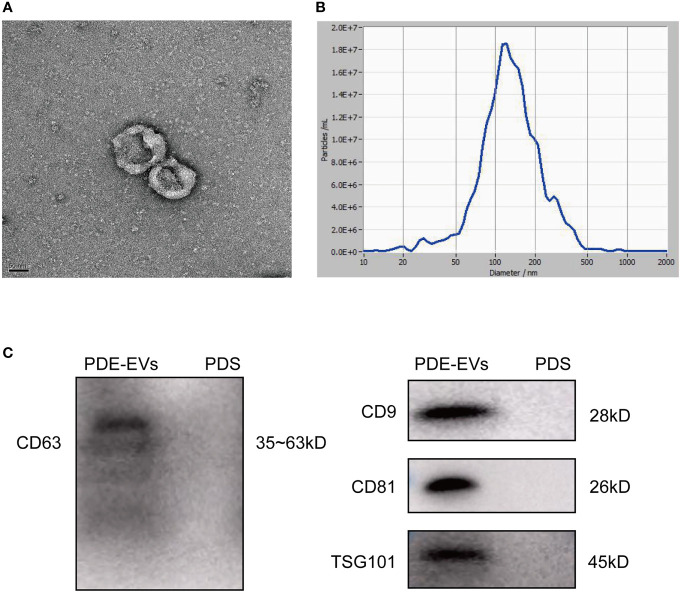
Characteristics of PDE-derived EVs. PDE-EVs were isolated from concentrated overnight PDE by differential ultracentrifugation. Unused 2.5% peritoneal dialysis solution (PDS) following ultracentrifugation was as a negative control. **(A)** The typical shape and morphology of PDE-EVs isolated by ultracentrifugation under TEM. **(B)** The diameters of PDE-EVs were measured by NTA. **(C)** Western blotting of PDE-EVs markers CD9, CD63, CD81, TSG101. *n* = 2. The results shown are from one representative of three independent experiments. TEM, Transmission electron microscopy; NTA, Nano tracking analysis.

EVs were isolated from concentrated overnight PDE samples by differential centrifugation. To identify the PDE-EVs, we visualized EVs with the typical rounded, cup-shaped bilayer morphology through TEM (**Figure 1A**). The size distribution of PDE-EVs was assessed by NTA. This analysis showed that the peak diameter of purified particles was approximately 120 nm, and that most were between 30 and 200 nm (**Figure 1B**). Western blotting analysis showed that the EVs from PDE expressed the endosome marker protein TSG-101 and tetraspanins CD63, CD9, and CD81 (**Figure 1C**). Taken together, our findings indicate that these vesicles are PDE-EVs.

### Proteomic Analysis Revealed the Potential Biomarkers in PDE-EVs From PD Patients With Different PSTRs

To systematically identify specific proteins capable of evaluating the PSTR, we collected PDE-EVs from 20 PD patients with different PSTRs and performed proteomics analysis. The demographic and clinical data of these patients are summarized in **Table 2**. Except for 4 h D/P_Cr_ and 24 h UF, no significant differences were found in the gender, age, dialysis time, eGFR, and Kt/V among patients with different peritoneal transport properties.

**Table 2:** Demographic and clinical characteristics of subjects in screening cohorts.

	H/A (n=10)	L/A (n=10)	*p-* Value
Gender (Male/Female)	6/4	5/5	0.653
Age (Year)	58.7 ± 14.82	59.1 ± 18.83	0.958
Dialysis Time (Month)	39.4 ± 22.68	40 ± 20.39	0.951
4h D/P_Cr_	0.78 ± 0.06	0.54 ± 0.05	<0.001^***^
eGFR (ml/min/1.73m^2^)	2.28 ± 2.49	2.04 ± 3.18	0.851
K*t/V* (ml/min)	2.09 ± 0.49	2.06 ± 0.62	0.901
24h UF (mL)	66.8 ± 594.5	736.6 ± 534.8	**0.016**

Values are means ± SD. eGFR, estimated glomerular filtration rate; Kt/V, urea clearance index; D/P_Cr_, dialysate/plasma creatinine ratio; UF, ultrafiltration volume; p < 0.05 was considered significant, and indicated in bold. ***p < 0.001, n = 10 per group.

**Figure 2:**
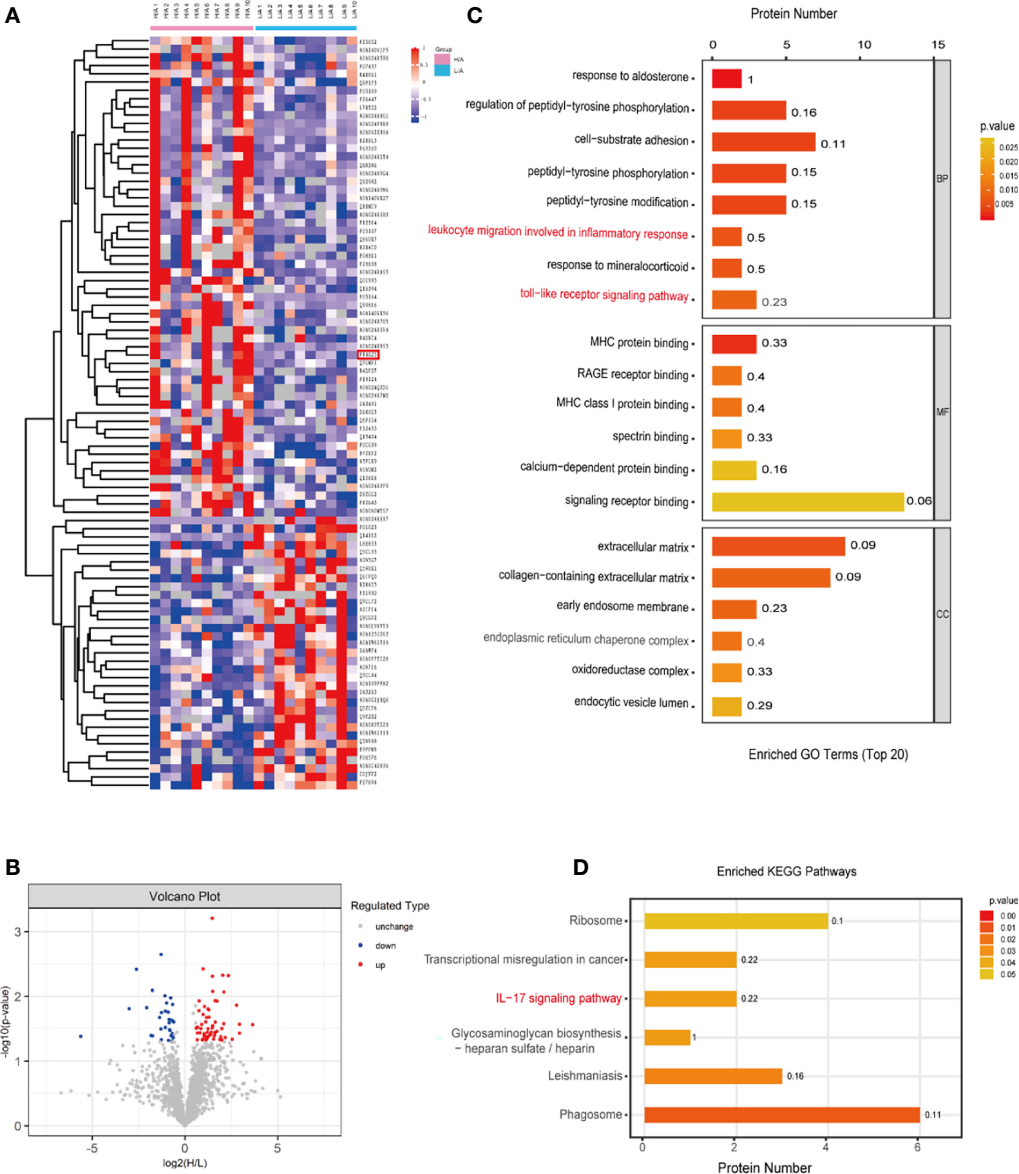
Different PDE-EVs protein profile. **(A)** The Heatmap showed the differentially expressed PDE-EVs proteins from the H/A group (*n* = 10) and the L/A group (*n* = 10). **(B)** Differentially regulated PDE-EVs proteins among all proteins detected in volcano plot based on fold change and *p*-value. **(C)** GO analysis for differentially expressed PDE-EVs proteins. **(D)** KEGG analysis for differentially expressed PDE-EVs proteins. BP, biological process; MF, molecular function; CC, cellular component.

The protein composition in PDE-EVs from patients with different PSTRs was determined using SP3 sample preparation combined with LC-MS/MS analysis. In total, 1859 proteins were identified in PDE-EVs, 91 of which were differentially expressed between the groups. Among them, the levels of 58 proteins were upregulated (*p <*0.05, fold change >1.5) and those of 33 were downregulated (*p <*0.05, fold change <0.6667) in the H/A group compared with the L/A group. Cluster analysis was performed for the differentially expressed proteins (**Figures 2A, B**). The most upregulated differentially expressed proteins identified in this study are shown in **Supplement 1**.

To explore the potential function of the identified differentially expressed proteins in PDE-EVs, we performed GO and KEGG functional enrichment analyses using Panther and Gene Ontology algorithms (**Figures 2C, D**). In the BP category, 91 differentially expressed proteins were related to cell-substrate adhesion, toll-like receptor signaling pathway, and leukocyte migration involved in the inflammatory response (**Figure 2C**). In the MF category, differentially expressed proteins related to RAGE receptor binding, MHC protein binding, MHC class I protein binding, calcium-dependent protein binding, and signaling receptor binding exhibited predominant enrichment (**Figure 2C**). Furthermore, the enriched KEGG pathways were related to phagosomes and the IL−17 signaling pathway (**Figure 2D**). We observed that the inflammatory response was enriched in all functional categories. Previous reports indicate that peritoneal injury triggers the activation of inflammatory cells, such as neutrophils, macrophages, and mesothelial cells, which recognize bacterial pathogens *via* Toll-like receptors (TLRs), activate relative inflammatory signaling pathways, and ultimately release a large number of pro-inflammatory cytokines (27–29). It is also suggested that higher PSTR may predispose patients to more inflammation than lower PSTR (30). Among the differentially expressed proteins between the groups, we identified three proteins (ITGB2, S100A8, and GP96) with a fold change ≥1.9 for their possible participation in the immune inflammatory response according to GO and KEGG analysis. Finally, we focused on GP96, because it is an obligate chaperone for TLRs (31) and its function may be involved in peritoneal inflammation.

### PDE-EVs Originated From Mesothelial Cells

**Figure 3:**
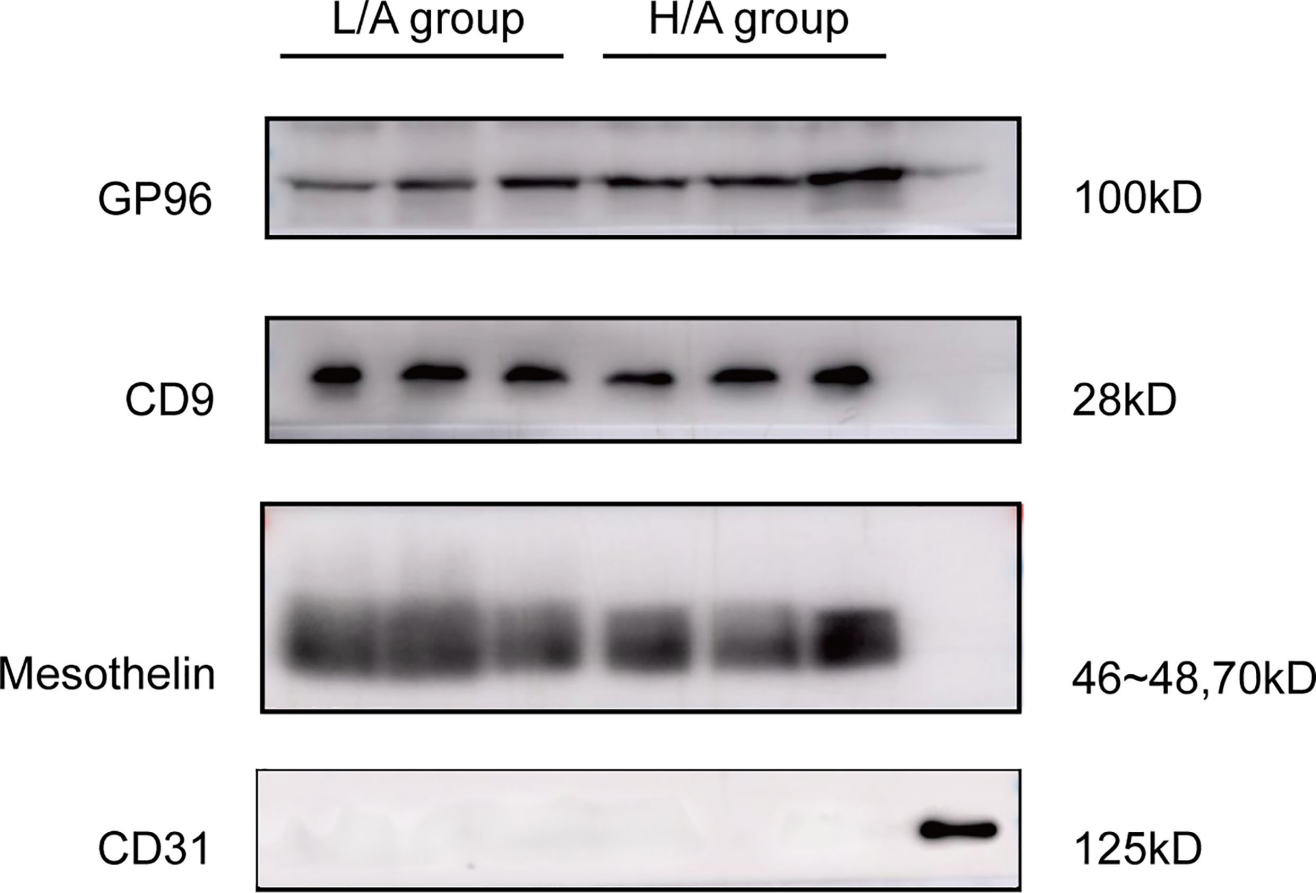
Validation of the origin of PDE-EVs and expression of target protein in PDE-EVs. Detection of EVs target protein GP96, CD9 (EVs marker), mesothelin (mesothelial marker), and CD31(endothelial marker) in PDE-EVs isolated from the H/A group and the L/A group. A lysate of human omentum was used as positive control for CD31. 50 µg of total protein from each sample was added to each well for western blotting; *n* = 7. The results shown are from one representative of three independent experiments.

To determine the origin of EVs in the PDE, PDE-EVs were isolated from concentrated overnight PDE samples from six PD patients by differential ultracentrifugation. Western blotting analysis of PDE-EVs revealed the presence of the mesothelial marker mesothelin and EV marker CD9, but not the endothelial marker CD31 (32) (**Figure 3**). These results show that at least a portion of PDE-EVs originated from mesothelial cells, which comprised the outer layer of the peritoneal lining, and were released into the peritoneal cavity.

### Validation of GP96 Expression Level in PDE-EVs

**Figure 4:**
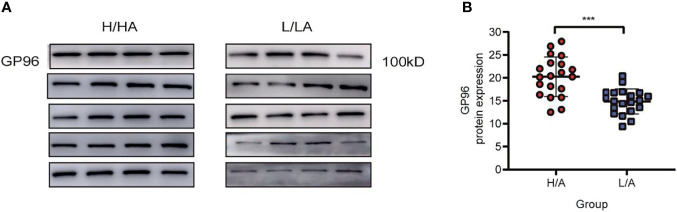
The relative expression level of PDE-EVs-GP96. **(A)** Western blotting analysis of PDE-EVs-GP96 expression in the H/A and the L/A group. 50 µg of total protein from each sample was added to each well. **(B)** Concentration of PDE-EVs-GP96 in the H/A and the L/A group was measured by ELISA. All data were presented as means ± SEM. Statistical analysis was performed with an unpaired Student’s *t*-test. ***p < 0.001, *n* = 20 per group.

To verify the differential expression of GP96 between the groups, western blotting and ELISA analyses were performed in a validation cohort (20 patients in each group). PDE-EVs were extracted from 500 mL of overnight PDE per sample. The clinical data of these patients are summarized in **Table 3**. No significant differences in age, gender, dialysis time, eGFR, Kt/V, and diabetes were observed between the H/A and L/A groups. However, there was a significant difference in 4 h D/P_Cr_ between the groups (*p <*0.001). In total, 50 µg of total protein from each sample was added to each well for western blotting. A band at 100 kDa corresponding to GP96 was detected in all samples, and this band had variable intensity between samples (**Figure 4A**). ELISA analysis revealed that the concentration of GP96 was significantly higher in the PDE-EVs of the H/A group than in those of the L/A group (*p <*0.001; **Figure 4B**), which confirmed our proteomic data.

**Table 3:** Demographic and clinical characteristics of subjects in validation cohorts.

	H/A (n=20)	L/A (n=20)	*p-Value*
Gender (Male/Female)	12/8	11/9	0.749
Age (Year)	59.65 ± 12.71	60.3 ± 11.92	0.868
Dialysis Time (Month)	50.5 ± 11.22	55.5 ± 12.65	0.194
4h D/P_Cr_	0.78 ± 0.062	0.58 ± 0.059	<0.001***
eGFR (ml/min/1.73m^2^)	2.88 ± 1.69	2.69 ± 1.34	0.698
K*t/V* (ml/min)	1.98 ± 0.37	1.87 ± 0.39	0.375
Diabetes (Yes/No)	7/13	4/16	0.288

Values are means ± SD. eGFR, estimated glomerular filtration rate; Kt/V, urea clearance index; p <0.05 was considered significant, and indicated in bold. ***p < 0.001, n = 20 per group.

### GP96 in PDE-EVs Was Associated With 4 h D/P_Cr_, UF Volume, and Inflammatory Indicators

**Figure 5:**
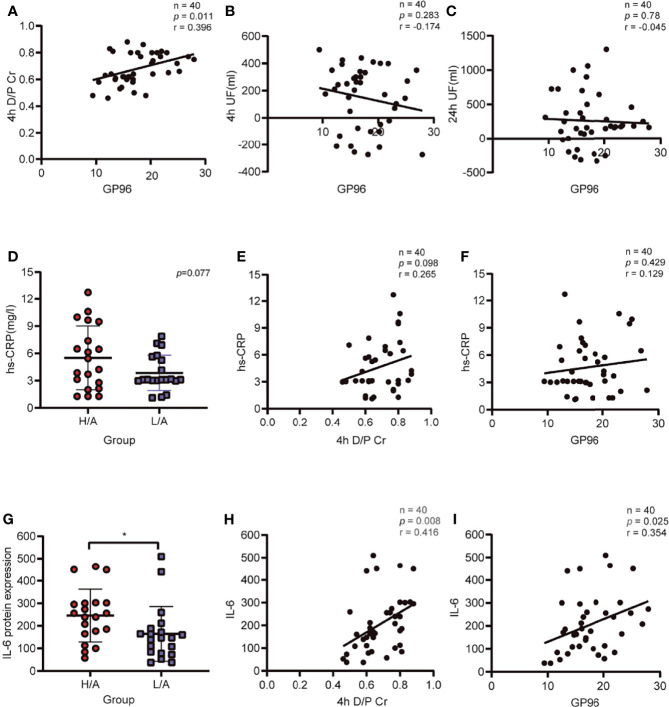
Correlation of PDE-EVs-GP96 with ultrafiltration (UF), the dialysate-to-plasma ratios of creatinine (D/P_Cr_), and inflammatory indicators. **(A)** PDE-EVs-GP96 positively correlated with 4h D/P_Cr_. **(B, C)** Relationship between PDE-EVs-GP96 levels and 4h UF or 24h UF. **(D)** Different levels of serum hs-CRP in the H/A and the L/A group. **(E, F)** Relationship between serum hs-CRP levels and 4h D/P_Cr_ or PDE-EVs-GP96 levels. **(G)** Different levels of dialysate IL-6 in the H/A and the L/A group. **(H, I)** Dialysate IL-6 levels positively correlated with 4h D/P_Cr_ or PDE-EVs-GP96 levels. All data are presented as means ± SEM. Statistical analysis was performed with an unpaired Student’s *t*-test, *p < 0.05, *n* = 20 per group. Spearman correlation was used for correlation analysis, *n* = 40, *p < 0.05.

As PDE-Evs-GP96 was increased in the H/A group, in combination with bioinformatics analysis, we hypothesized that GP96 in PDE-EVs might be correlated with 4 h D/P_Cr_, UF volume, and inflammatory indicators. The expression level of GP96 in PDE-EVs was positively correlated with 4 h D/P_Cr_ (*r* = 0.396, *p <*0.05), whereas the correlation between 4 h UF or 24 h UF with GP96 in PDE-EVs was not significant (**Figures 5A–C**).

As an indicator of systemic inflammation, serum hs-CRP levels were not significantly different between the groups (*p* = 0.077; **Figure 5D**), and there was no correlation with 4 h D/P_Cr_ or the levels of GP96 in PDE-EVs (**Figure 5E, F**). However, the local intraperitoneal inflammatory index showed that the IL-6 level in PDE was significantly higher in the H/A group than in the L/A group (*p <*0.05; **Figure 5G**), and IL-6 levels were significantly positively correlated with D/P_Cr_ (*r* = 0.416, *p <*0.05; **Figure 5H**). We also observed that the level of GP96 was positively correlated with the level of intraperitoneal IL-6 levels (*r* = 0.354, *p <*0.05; **Figure 5I**). Taken together, these results indicate that the level of PDE-EVs-GP96 is associated with 4 h D/P_Cr_ and the intraperitoneal inflammatory condition of PD patients.

### GP96-Enriched PED-EVs Promoted Secretion of Proinflammatory Cytokines in Macrophages

**Figure 6:**
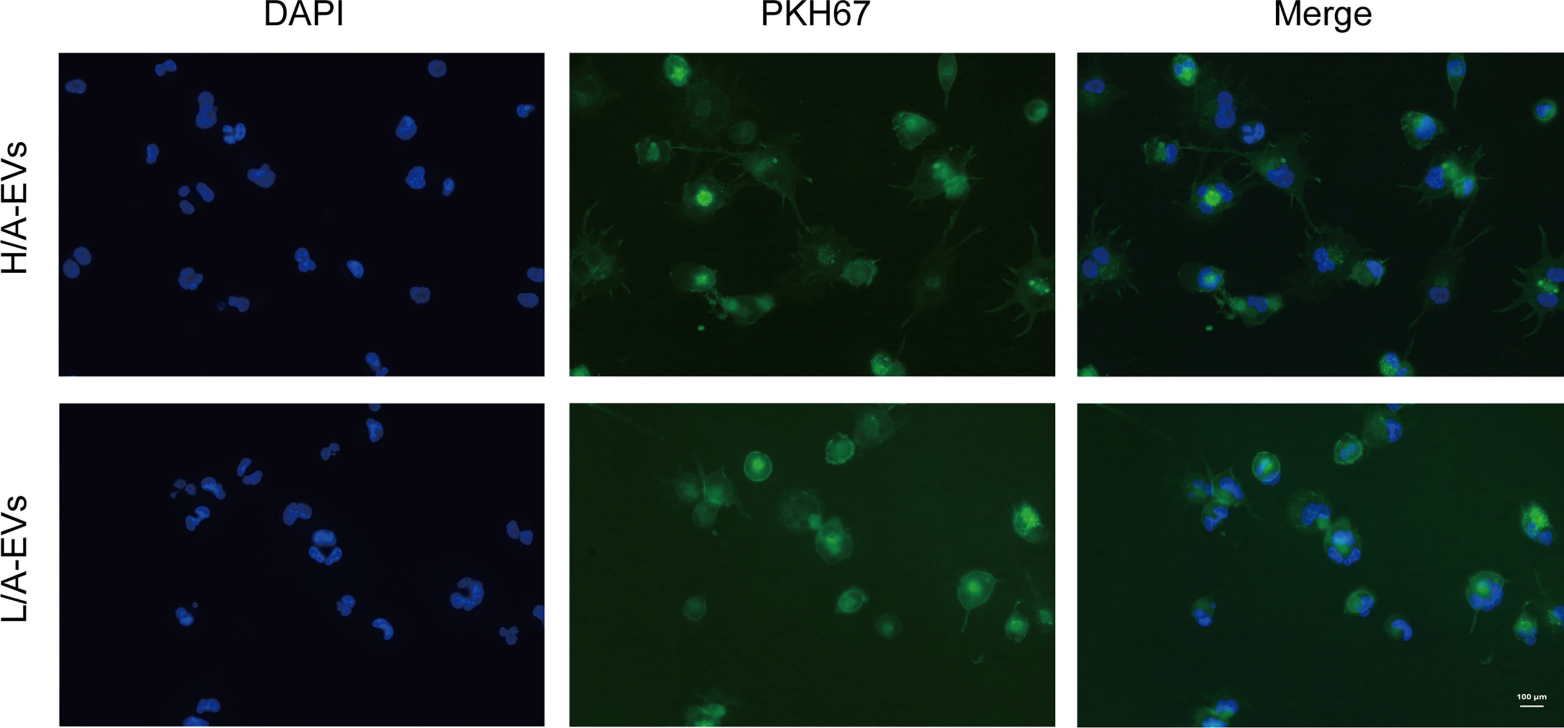
Uptake of PDE-EVs by macrophages. PDE-EVs from the H/A group or the L/A group labeled with PKH67 (green) were separately added to the culture of macrophages for 24 h. The labeled EVs were distributed around the DAPI-stained nuclei (blue). This colocalization was determined using fluorescence microscopy. The results shown are from one representative of three independent experiments. DAPI, 4′,6-diamidino-2-phenylindole.

Peritoneal macrophages are at the front line of the host’s peritoneal defense against external pathogens in PD patients (9, 11). As phagocytosis is one of the primary functions of macrophages, we assessed the ability of macrophages to take up PDE-EVs. PDE-EVs from different groups labeled with PKH67 (green) were separately added to the culture of macrophages. The labeled EVs were distributed around the DAPI-stained nuclei (blue). These results clarified that both PDE-EV types can be phagocytized and, thus, potentially regulate the function of macrophages (**Figure 6**).

**Figure 7:**
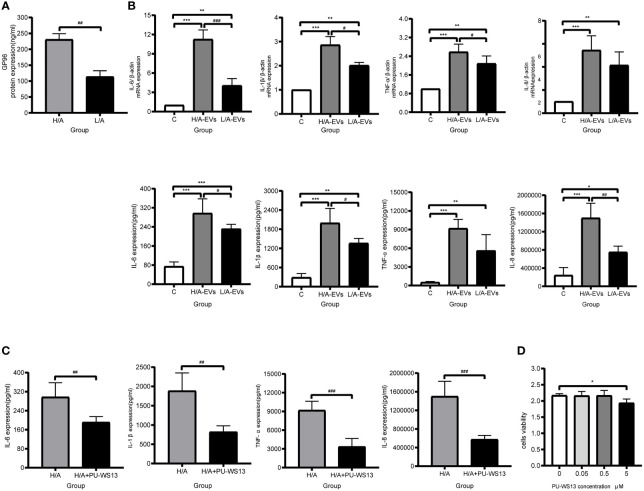
PDE-EVs modulate inflammation *via* GP96. **(A)** Concentration of PDE-EVs-GP96 in the H/A and the L/A group was measured by ELISA (*n* = 4 per group). **(B)** Relative mRNA or protein expressions of inflammatory factors IL-6, IL-1β, TNF-α, IL-8 in macrophages were measured by real-time PCR or ELISA. *In vitro*, macrophages were incubated with H/A-PDE EVs (H/A-EVs, 2 mg/ml), L/A-PDE EVs (L/A- EVs, 2 mg/ml) (*n* = 4). PBS was added in control group. **(C)** By using specific inhibitor PU-WS13, inhibition of GP96 reduced the production of pro-inflammatory cytokines induced by PDE-EVs. Macrophages were stimulated with PDE-EVs isolated from the H/A group (2 mg/mL) for 24 hours alone or in combination with PU-WS13 (0.5 μM) before PDE-EVs for 1 hour (n = 4 per group). H/A group were treated with H/A-PDE EVs and DMSO. Culture supernatant was evaluated for secreted IL-6, IL-1β, TNF-α and IL-8 by ELISA. **(D)** The viability of macrophages was determined by measuring the absorbance at 450nm through a microplate reader. Macrophages were stimulated with different concentration of PU-WS13 for 24 h (n = 6 per group). Data represent mean ± SEM of four independent experiments. Statistical analysis was performed with an unpaired Student’s *t*-test for two groups or one-way analysis of variance (ANOVA) for three groups. *p < 0.05, **p < 0.01 and ***p < 0.001, compared with the C group or 0 group; ^#^ *p* < 0.05, ^##^ *p* < 0.01 and ^###^ *p* < 0.001 compared with the H/A group or H/A-EVs group.

The control group (C), PDE-EVs from the H/A group (H/A-EVs), and PDE-EVs from the L/A group (L/A-EVs) were used to compare the immunomodulatory effects of PDE-EVs from both groups. To attain PDE-EVs containing >100 ng/mL GP96 to activate macrophages, based on previous studies (33, 34), EVs from 3 L of overnight PDE on 2 consecutive days per sample were isolated. We also found that the GP96 level was significantly higher in the H/A group than in the L/A group (*p* = 0.0015; **Figure 7A**). During 24 h of incubation, secretion of the proinflammatory mediators TNF-α, IL-8, IL-6, and IL-1β mRNA was significantly increased in the presence of PDE-EVs (*p <*0.01; **Figure 7B**). However, the levels of IL-6, IL-1β, and TNF-α mRNA were significantly higher in PDE-EVs from the H/A group than in those from the L/A group (*p <*0.05; **Figure 7B**). ELISA analysis further revealed that the concentrations of IL-6, IL-1β, and IL-8 in the culture supernatants were upregulated after H/A PDE-EVs treatment (p <0.05; **Figure 7B**).

Next, we assessed whether macrophages treated with a GP96-specific inhibitor could reduce proinflammatory response. Macrophages that were stimulated with H/A PDE-EVs were treated with 0.5 µM PU-WS13. PU-WS13-treated macrophages experienced a significant decline in the levels of proinflammatory molecules IL-6, IL-1β, TNF-α, and IL-8 induced by H/A PDE-EVs (*p <*0.01; **Figure 7C**). To eliminate the possibility that the observed anti-inflammatory impacts of PU-WS13 were due to cell death, we measured the overall viability of macrophages treated with 0.5 µM PU-WS13 for 24 h, and found no differences compared with macrophages treated with the vesicle (*p* >0.05; **Figure 7D**).

Taken together, these data suggest that PDE-EVs from patients with different PSTRs are taken up by macrophages and that they affect the expression of proinflammatory cytokines *via* GP96.

### A GP96 Inhibitor Suppressed The PDF-Induced Inflammatory Response in Uremic Peritoneal Dialysis Rats

**Figure 8:**
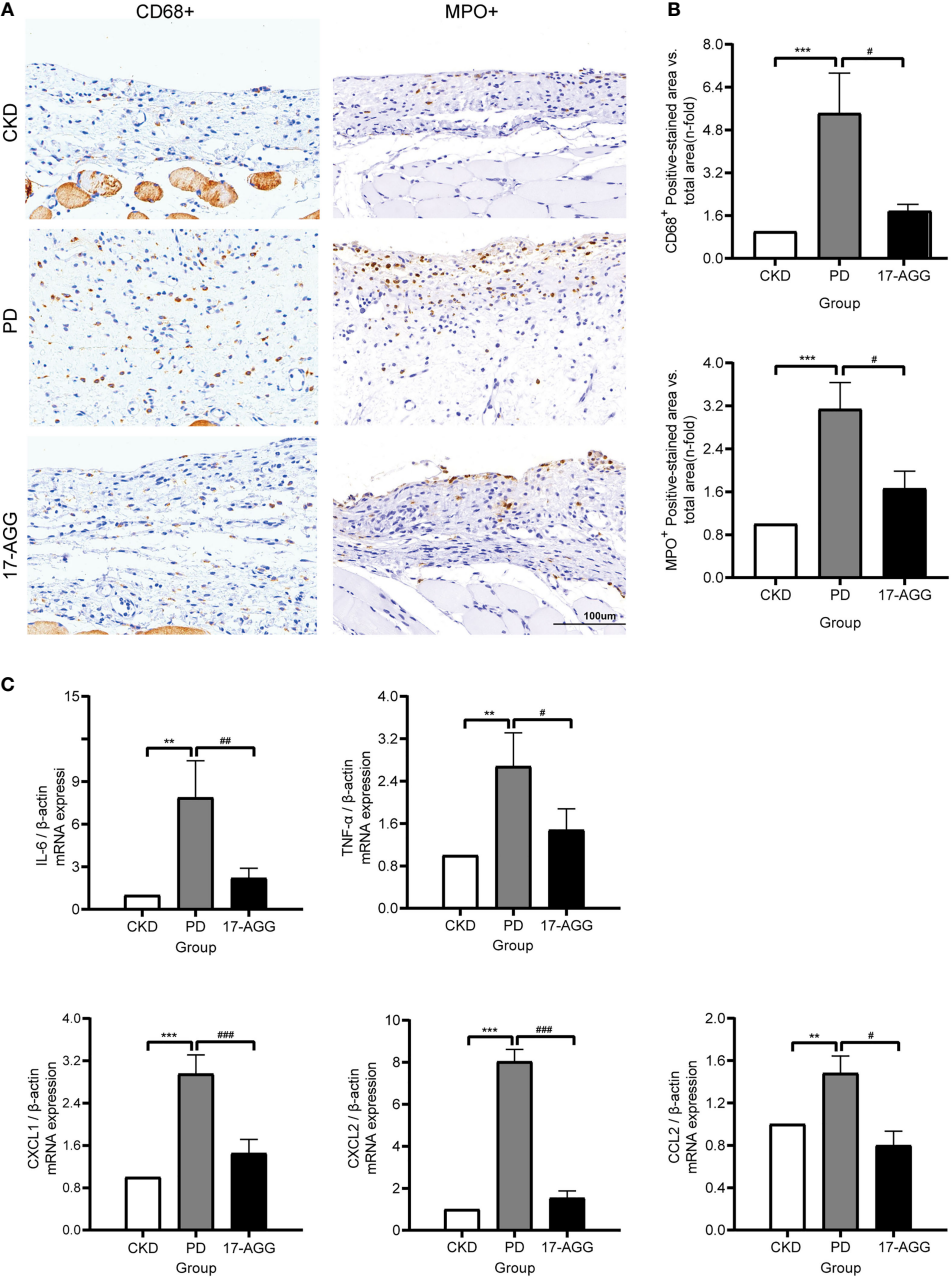
A GP96 inhibitor suppressed the PDF-induced inflammatory response in uremic peritoneal dialysis rats. SD rats underwent a subtotal nephrectomy. Four weeks after subtotal nephrectomy, the rat underwent surgery to implant a peritoneal catheter for the acute model of exposure to 4.25% Dianeal or saline (5 mL/100 g body weight, twice a day for 10 days). The PD group was instilled with PDF. For 17-AGG experiments, PDF-instilled rats were treated with 17-AGG (50 mg/kg) every 3 days *via* the peritoneal catheter, starting 2 h before daily PDF instillation. Daily instillation of PDF caused a marked increase in the infiltration of inflammatory cells and proinflammatory gene expression in the peritoneum, which was reversed by 17-AGG. **(A)** Immunohistochemical staining of CD68^+^ or MPO^+^ in the peritoneum sections from CKD, PD and 17-AGG groups, respectively. A representative picture of each group (magnification ×200) is shown. Scale bar: 100 μm. **(B)** The quantification of the immunostaining. **(C)** Real-time PCR of gene expression. All results were presented as mean ± SD from three independent experiments. Statistical analysis was performed with one-way analysis of variance (ANOVA) for three groups. **p < 0.01 and ***p < 0.001, compared with the CKD group; ^#^ *p* < 0.05, ^##^ *p* < 0.01 and ^###^ *p* < 0.001 compared with the PD group; *n* = 5 per group.

To verify the biological roles of GP96 in peritoneal inflammatory pathogenesis, a GP96 inhibitor (17-AGG) was applied to a rat model of peritoneal dialysis. 17-AGG is not a pharmacological GP96-specific inhibitor, but it binds to GP96 in the ATP-binding site, thereby inhibiting its ATPase activity (35). To assess local inflammatory cell recruitment in the peritoneum, macrophage (CD68+ cells) and neutrophil (MPO^+^ cells) infiltration was evaluated by immunohistochemistry (IHC). Daily instillation of PDF caused a marked increase in macrophage and neutrophil infiltration in the peritoneum of the PD group (*p <*0.001, **Figures 8A, B**). We also analyzed the expression of the inflammatory molecules IL-6, TNF-α, CXCL1, CXCL2, and CCL2 in the peritoneal membrane of animals in each experimental group by real-time PCR. PDF also promoted a significant increase in the expression of these inflammatory mediators (*p <*0.01, **Figure 8C**). However, 17-AGG infusions effectively attenuated infiltration of macrophages or neutrophils into the peritoneum and downregulated the mRNA levels of proinflammatory factors (*p <*0.05, **Figures 8A–C**). These data confirmed that GP96 participated in inflammation during peritoneal dialysis, supporting its potential as a therapeutic target to alleviate peritoneal inflammation.

## Discussion

In the present study, we found that the expression levels of GP96 in EVs from PDE were significantly higher in patients with high PSTRs and positively correlated with the PSTR and dialysate IL-6 concentration. We verified that GP96-enriched EVs stimulated macrophages to secrete more proinflammatory cytokines, and that the GP96 inhibitor attenuated local inflammatory cell infiltration and proinflammatory gene expression in the peritoneum of a rat peritoneal dialysis model.

MicroRNAs or proteins in EVs are promising biomarkers for several diseases and pathologic states (21, 36), and PDE is a non-invasive and easily collected sample. To the best of our knowledge, this study is the first one to report that PDE-EVs from PD patients with different peritoneal membrane transport rates have different proteomic profiles. These data can be used to identify possible novel biomarkers of PSTR and the peritoneal inflammatory state. Proteomic analysis of 91 proteins showed differential expression between high transporters (H/A) and low transporters (L/A). Only a few proteins identified in the present study overlapped with those found in previous studies on the proteomic profile of PDE obtained from different transporters, such as complement factor C4A (22, 23). This suggests that the content of EVs is highly specialized for particular biological and pathological conditions (13, 14). The presence of mesothelin suggested that at least a portion of EVs in the PDE originated from mesothelial cells, which is in agreement with previous findings (18, 19). Peritoneal mesothelial cells are the most important constituents of the peritoneum, and are predominantly involved in the regulation of the intraperitoneal homeostasis of the abdominal cavity by releasing a number of inflammatory mediators to govern immune surveillance, initiate tissue repair, regulate inflammatory response, and control fluid and solute transport (37). Thus, mesothelium-derived EVs may carry special cargos to trigger signals that cause peritoneal damage. Although we did not detect the presence of the endothelial marker CD31, PDE-EVs may still originate from endothelial cells and other sources (i.e. monocytes and fibroblasts), and the origin of these EVs should be further investigated in the future.

Proteins related to EVs endocytosis and the immune response are enriched in EVs from high transporters. Some of these proteins, such as heat shock proteins (HSPs) and S100 proteins, are involved in exosome-mediated immunoregulation or inflammation (38, 39). Several differentially expressed proteins in the present study are associated with the TLR signaling pathway, leukocyte migration involved in the inflammatory response, and the IL−17 signaling pathway, and these pathways participate in peritoneal inflammation (27, 40). Changes in the expression levels of these proteins in PDE-EVs are likely to originate from an inflammatory background. Consistent with our findings, several studies have suggested that the high transporter peritoneal membrane characteristics are associated with the inflammatory status, with increased levels of inflammatory markers present in the PDE and macrophages/mononuclear cell infiltration in the peritoneum of the high transporters (8–11). We, therefore, assumed that PDE-EVs may participate in peritoneal inflammatory injury through these candidate proteins.

To identify the role of proteins in PDE-EVs during peritoneal inflammation, we screened the proteins that were differentially expressed between the groups. We focused on GP96, which is upregulated in EVs derived from the PDE of PD patients. GP96, encoded by the HSP90B1, is an endoplasmic reticulum (ER)-associated chaperone of HSP90. Additionally, GP96 is constitutively expressed in virtually every cell type, and its expression is upregulated by cytosolic and ER stress, which is caused by several factors, including the accumulation of misfolded proteins, oxidative stress, and acidosis (41), some of which are often found in the peritoneal microenvironment of PD patients. Recently, it has been shown that GP96, which is induced by ER stress in inflammatory lesions, participates in autoimmune and inflammatory diseases, such as systemic lupus erythematosus, rheumatoid arthritis, and sepsis (33, 42, 43). Local and subclinical inflammatory reactions could lead to progressive peritoneal injury and changes in the PSTR (6). The level of the intraperitoneal inflammation marker, dialysate IL-6, is the most reliable predictor of PSTR (10). Our data showed that the levels of EV-GP96 were significantly higher in PD patients with high PSTRs. In addition, the concentration of PDE-EV-GP96 was positively correlated with PSTR and dialysate IL-6 levels. Therefore, PDE-EV-GP96 is directly related to the inflammatory state of the peritoneum and the PSTR. Our study was a single-center, cross-sectional study. Thus, further prospective cohort studies are needed to determine the sensitivity and specificity of PDE-EVs-GP96 as a biomarker for evaluating the PSTR. In addition, whether PDE-EVs-GP96 affects the rate of PD technical failure, peritoneal dysfunction, and survival rate of PD patients also needs to be studied in detail in the future.

GP96 is an obligate chaperone for TLRs and multiple integrins, and plays a critical role in regulating both innate and adaptive immunity (31, 41). In the absence of obvious infection or pathogens, GP96 is a molecular trigger for inflammation when it leaves the ER and appears on the surface or outside cells (44). Bioinformatic analysis showed that EVs-GP96 participated in the TLR signaling pathway, which was involved in inflammation induced either by recurrent peritoneal inflammation during PD or prolonged exposure to PD solution (27, 45).

EVs have an inherent signaling capacity that is important for the global inflammatory response (46). EVs from body fluids more closely reflect the *in vivo* situation than EVs from cell culture (46). Hence, in the present study, we used GP96-enriched PDE-EVs from patients with high PSTRs to treat macrophages and found that these EVs could be internalized by macrophages, resulting in the increased secretion of proinflammatory molecules, including IL-6, IL-1β, TNF-α, and IL-8. The pharmacological GP96-specific inhibitor, PU-WS13, could reverse the effect induced by GP96-enriched PDE-EVs, which was consistent with other studies (47–49). A number of studies have elucidated the possible mechanisms by which GP96 can activate antigen-presenting cells *via* the TLR2/4 pathway, which causes the activation of inflammation-related transcription factors, including NF-κB, or NLRP3 inflammasome signaling, which promotes the expression of proinflammatory chemokines and cytokines (31, 33, 44, 50, 51). Further investigations are required to identify the signaling pathways that GP96 triggers in recipient cells to induce peritoneal inflammation.

GP96 levels are increased in peritoneal mesothelial cells isolated from PD patients (52). Thus, we explored the potential effects of pharmacological GP96 inhibitors on peritoneal inflammation in a PD rat model. In accordance with previous studies (49, 53), intraperitoneal injection of GP96 inhibitor significantly decreased the number of immune-infiltrating cells in the rat peritoneum and downregulated the gene expression of inflammatory cytokines (IL-6 and TNF-α) and chemokines (CCL2), including the neutrophil-related CXCL1/CXCL2. Our results suggest that GP96 is involved in PD-related peritoneal inflammation and may be a new therapeutic target. However, the GP96 inhibitor we used here, 17-AGG, has an intracellular effect on GP96 ATPase activity but does not target EV-GP96. Hence, we were unable to determine the role of EV-96 *in vivo*, which requires improved research methods.

In conclusion, the present study identified a unique profile of EV proteins in PDE from patients with different PSTRs. We found that the expression level of PDE-EV-GP96 was higher in PD patients with high PSTRs, and was positively correlated with the PSTR and intraperitoneal IL-6 levels. GP96 protein in EVs could initiate an inflammatory response by stimulating the activation of macrophages and release of inflammatory cytokines *in vitro*. Our findings provide evidence that PDE-EV-GP96 has the potential to be an indicator to evaluate the status of peritoneal inflammation and PSTR in PD patients. We also reaffirm that EVs as extracellular mediators play an important role in the pathophysiology of peritoneal inflammation. In addition, the findings of this study contribute to a deeper understanding of PDE-EVs-proteins as potential therapeutic targets, and provide insights and solutions for local peritoneal inflammation and UF failure.

## Data Availability Statement

The original contributions presented in the study are publicly available. This data can be found here: NODE, OEP003072, link: https://www.biosino.org/node/project/detail/OEP003072.

## Ethics Statement

The studies involving human participants were reviewed and approved by the Ethics Committee of Shanghai Ninth People’s Hospital, Shanghai Jiao Tong University School of Medicine (SH9H-2020-T23-1). The patients/participants provided their written informed consent to participate in this study. The animal study was reviewed and approved by the Animal Care and Use Committee of the School of Medicine, Shanghai Jiao Tong University (HKDL [2018] 38). Written informed consent was obtained from the individual(s) for the publication of any potentially identifiable images or data included in this article.

## Author Contributions

FD and YL designed the research. JF and YT conducted the research and analyzed the results. JF wrote the paper. OJ and SW conducted the real-time PCR experiments and analyses. ZC, AS, and PL performed the western blotting measurements and analyses. YZ, HZ, and HR conducted the proteomic analysis. All the authors read and approved the final manuscript.

## Funding

This work was sponsored by the Shanghai Health Commission Clinical research project (202140430), the National Natural Science Foundation of China (8217030178), Research Program of 9th People’s Hospital affiliated to Shanghai Jiao Tong University School of Medicine (JYLJ2018011) and Fundamental Research Program Funding of 9th People’s Hospital affiliated to Shanghai Jiao Tong University School of Medicine (JYZZ058).

## Conflict of Interest

YZ, HZ, and HR were employed by Shanghai Applied Protein Technology Co., Ltd.

The remaining authors declare that the research was conducted in the absence of any commercial or financial relationships that could be construed as a potential conflict of interest.

## Publisher’s Note

All claims expressed in this article are solely those of the authors and do not necessarily represent those of their affiliated organizations, or those of the publisher, the editors and the reviewers. Any product that may be evaluated in this article, or claim that may be made by its manufacturer, is not guaranteed or endorsed by the publisher.
