# Supplementary material for: Glycoprotein 96 in Peritoneal Dialysis Effluent-Derived Extracellular Vesicles: A Tool for Evaluating Peritoneal Transport Properties and Inflammatory Status
Source: Front Immunol. 2022 Feb 10;13:824278. doi: 10.3389/fimmu.2022.824278 (PMC8866190; doi:10.3389/fimmu.2022.824278)
Supplement: Supplementary file 1 [file Table_1.pdf]

**Supplement 1**. The most upregulated differentially expressed protein in PDE-EVs of the H/A group

| Protein accession | Protein name | Fold of change(H/L) | *p*-value |
|---|---|---|---|
| P32455 | Guanylate-binding protein 1 | 12.64 | 0.0276 |
| A0A024F8R8 | HLA-DRB1 | 7.73 | 0.0371 |
| P05164 | Myeloperoxidase | 7.72 | 0.0272 |
| P05107 | Integrin beta-2 | 6.91 | 0.0138 |
| A0A0S2Z3S6 | Cytochrome b-245 beta polypeptide isoform 1 | 5.87 | 0.0462 |
| B4DP27 | Transmembrane emp24 domain-containing protein 2 | 5.09 | 0.0048 |
| P12314 | High affinity immunoglobulin gamma Fc receptor I | 4.54 | 0.0435 |
| A0A024QZD1 | Ribosomal protein L18 | 4.33 | 0.0086 |
| A0A024R8U1 | Solute carrier family 16 (Monocarboxylic acid transporters), member 3, isoform CRA_ a | 4.27 | 0.0266 |
| Q86UX7 | Fermitin family homolog 3 | 4.11 | 0.0047 |
| A0A024R7M0 | Transmembrane emp24 protein transport domain containing 9, isoform CRA_a | 3.99 | 0.0316 |
| Q8N386 | Leucine-rich repeat-containing protein 25 | 3.97 | 0.0475 |
| Q99816 | Tumor susceptibility gene 101 protein | 3.83 | 0.0422 |
| A0A0A0MTS7 | Titin | 3.70 | 0.0468 |
| B2R4C0 | 60S ribosomal protein L18a | 3.36 | 0.0119 |
| B2RBL3 | Thymidine phosphorylase | 3.31 | 0.0347 |
| P62269 | 40S ribosomal protein S18 | 3.12 | 0.0252 |
| A0A024R705 | Vacuolar protein sorting-associated protein 4A | 3.09 | 0.0365 |
| A0A140VJP5 | S-adenosylmethionine synthase isoform type-2 | 3.09 | 0.0363 |
| P28838 | Cytosol aminopeptidase | 3.08 | 0.0117 |
| D6RGG3 | Collagen alpha-1(XII) chain | 3.00 | 0.0409 |
| P05109 | Protein S100-A8 | 2.94 | 0.0461 |
| Q15404 | Ras suppressor protein 1 | 2.83 | 0.0085 |
| P08311 | Cathepsin G | 2.83 | 0.0286 |
| P18124 | 60S ribosomal protein L7 | 2.82 | 0.0049 |
| B7ZKY2 | Peripheral plasma membrane protein CASK | 2.79 | 0.0006 |
| A0AUM2 | FOLR2 protein (Fragment) | 2.78 | 0.0308 |
| A0A024RAS5 | Rho GDP dissociation inhibitor (GDI) beta, isoform CRA_a | 2.68 | 0.0357 |
| A0A024R9G4 | Family with sequence similarity 49, member B, isoform CRA_a | 2.61 | 0.0386 |
| A0A024RDT4 | Lymphocyte cytosolic protein 1 (L-plastin), isoform CRA_a | 2.53 | 0.0403 |
| Q92982 | Ninjurin-1 | 2.48 | 0.0211 |
| A5PLK9 | Bone morphogenetic protein 1 | 2.46 | 0.0163 |
| B4DXC4 | Atlastin-3 | 2.39 | 0.0157 |

| | | | |
|---|---|---|---|
| A0A024R1V4 | 60S ribosomal protein L27 | 2.38 | 0.0421 |
| A0A024R9N6 | EH-domain containing 4, isoform CRA_a | 2.38 | 0.0318 |
| P26447 | Protein S100-A4 | 2.32 | 0.0362 |
| L7RT22 | Integrin beta;Integrin beta-5 | 2.28 | 0.0447 |
| Q1RMC9 | ERBB2IP protein | 2.24 | 0.0213 |
| K4RH61 | Matrix metalloproteinase-14 | 2.21 | 0.0473 |
| A0A024R2F9 | Transmembrane protein 43 | 2.19 | 0.0250 |
| A0A140VK56 | cDNA FLJ61307, highly similar to Netrin G1 | 2.18 | 0.0423 |
| A0A024R183 | Cyclin-dependent kinase 16, partial [Neogale vison] | 2.08 | 0.0143 |
| Q6FIG4 | RAB1B protein | 2.06 | 0.0412 |
| A0A140VK27 | Leukotriene A(4) hydrolase | 2.06 | 0.0378 |
| A0A024R8S5 | Protein disulfide-isomerase | 2.05 | 0.0404 |
| Q15818 | Neuronal pentraxin-1 | 1.98 | 0.0038 |
| P14625 | Glycoprotein 96 | 1.97 | 0.0192 |
| Q9P1F3 | Costars family protein ABRACL | 1.96 | 0.0463 |
| P0CG39 | POTE ankyrin domain family member J | 1.92 | 0.0470 |
